# Intrinsic Tryptophan Fluorescence in the Detection and Analysis of Proteins: A Focus on Förster Resonance Energy Transfer Techniques

**DOI:** 10.3390/ijms151222518

**Published:** 2014-12-05

**Authors:** Amar B. T. Ghisaidoobe, Sang J. Chung

**Affiliations:** Department of Chemistry, Dongguk University, Seoul 100-715, Korea; E-Mail: shvaki@live.com

**Keywords:** FRET, label free detection, tryptophan fluorescence, intrinsic fluorescence, protein imaging, biosensors, immunoassay

## Abstract

Förster resonance energy transfer (FRET) occurs when the distance between a donor fluorophore and an acceptor is within 10 nm, and its application often necessitates fluorescent labeling of biological targets. However, covalent modification of biomolecules can inadvertently give rise to conformational and/or functional changes. This review describes the application of intrinsic protein fluorescence, predominantly derived from tryptophan (λ_EX_ ∼ 280 nm, λ_EM_ ∼ 350 nm), in protein-related research and mainly focuses on label-free FRET techniques. In terms of wavelength and intensity, tryptophan fluorescence is strongly influenced by its (or the protein’s) local environment, which, in addition to fluorescence quenching, has been applied to study protein conformational changes. Intrinsic Förster resonance energy transfer (iFRET), a recently developed technique, utilizes the intrinsic fluorescence of tryptophan in conjunction with target-specific fluorescent probes as FRET donors and acceptors, respectively, for real time detection of native proteins.

## 1. Introduction

Fluorescence spectroscopy has become a crucial tool in biochemical research by virtue of its robustness, high sensitivity and non-invasiveness [1]. The continuous development of sophisticated optics and electronics has stimulated the use of fluorescent moieties (fluorophores) for most biochemical analysis in preference to expensive and difficult to handle radio active tracers [2]. Fluorophores absorb light of a specific wavelength (λ_EX_), and after a brief interval, termed the fluorescence lifetime (𝜏), energy is emitted at a longer and specific wavelength (λ_EM_) [3]. In general, the fluorescence study of biomolecules, e.g., lipids, (oligo)saccharides, oligonucleotides (DNA and RNA), proteins and membranes, requires elaborate fluorescent labeling processes. A large variety of fluorescent molecules are currently (commercially) available, which include biological fluorophores (e.g., the green fluorescent protein), organic dyes (e.g., fluorescein) and fluorescent nanoparticles (e.g., quantum dots). The choice of a fluorophore mainly depends on the photophysical properties (e.g., absorption and emission wavelength, Stokes shift and quantum yield) applicable for specific research purposes and detection techniques. Another aspect which may influence the selection of a fluorescent moiety is the ease and selectivity at which it can be integrated in a specific target without impeding the natural function thereof. In this vein, especially the green fluorescent protein (GFP) and its derivatives have found broad application in biochemistry and cell biology. GFP, a natural protein, was first isolated by Shimomura *et al.* from *Aequorea* jellyfish, and its encoding gene can be expressed in other organisms to give functional (chimeric) GFP [4,5]. Numerous approaches have been developed to selectively introduce the more versatile organic dyes into (purified) proteins via chemical recognition-based labeling [6]. Furthermore, the use of biomimetics, such as fluorescent nucleotide triphosphates and fluorescent amino acid derivatives as monomers in the biosynthesis of their respective polymers, represents a viable approach to incorporate fluorescent tags.

Once the appropriate fluorescence source is installed, the photophysical properties of the target can be analyzed with four different types of instruments, which individually provide distinct information. First, spectrofluorometers and microplate readers are used to measure the average photophysical properties of bulk samples ranging from µL to mL quantities. Recently, it was demonstrated that the fluorescence readout of biological assays can be accomplished with a smartphone-based fluorimeter, which offers a new approach towards portable biomolecular fluorescence assays [7]. Secondly, fluorescence microscopy can be used to resolve fluorescence as a function of spatial coordinates in two or three dimensions for objects less than *∼*100 nm in size, and this gave rise to the development of single molecule fluorescence spectroscopy [8]. Next, fluorescence scanners (including microarray readers) are employed for macroscopic objects, such as electrophoresis gels and chromatograms, and can resolve fluorescence as a function of spatial coordinates in two dimensions. Finally, flow cytometers allow fluorescence analysis of individual particles (usually cells) as they flow in a fluid stream.

One of the major shortcomings of fluorescent molecules is their photolability, resulting in their irreversible degradation or photobleaching. The rate of photobleaching depends on several factors, such as the fluorophore environment and the excitation wavelength. The development of new excitation methods using two-photon and even three-photon excitation (e.g., in the case of two-photon excitation, fluorophores that have an absorption maximum of 300 nm are excited with two photons of light with a wavelength of 600 nm) can limit the rate of photobleaching [9,10]. Utilizing the different fluorescence instruments, a plethora of fluorescence analysis methods have been developed to image complex biomolecular assemblies, such as protein-protein and protein-ligand interactions, to study protein conformations and for (cell-based) assays. Förster resonance energy transfer (FRET), often used as a molecular ruler to determine intra-/inter-molecular distances between a donor fluorophore and a acceptor chromophore, has emerged as a versatile and powerful fluorescence technique [11].

Although significant progress has been achieved in the fluorescent labeling of biomolecules and the application thereof, several major drawbacks still remain. The often tedious and time-consuming procedures encountered during labeling of the targets may lead to unforeseen complications. It is well known that the covalent modification of biological targets can result in structural and functional changes thereof. To avoid this drawback, many approaches utilize the intrinsic fluorescence exhibited by most cells, *i.e.*, auto-fluorescence, originating from a large collection of species, including structural components e.g., proteins, flavins, NAD(P)H and metabolites [12]. The autofluorescence spectra of cells are usually broad, encompass most of the visible spectral range and can interfere with fluorescence measurements [13]. In particular, the intrinsic fluorescence of proteins, originating from the aromatic amino acid constituents, have been extensively explored to study protein dynamics and conformational changes. A myriad of reports deals with changes in fluorescence intensity, absorption and emission maximum (λ*_max_*), band shape, anisotropy and fluorescence lifetimes of the fluorescent amino acid residues in native proteins [14].

This review aims to provide insight into the utilization of tryptophan (Trp) fluorescence, the dominant source of intrinsic protein fluorescence. Here, a brief description of the prevailing methods that utilize the intrinsic fluorescence of Trp residues in (native) target proteins is presented. These methods include analysis of Trp fluorescence properties, such as changes in emission wavelength and intensity, absorption maxima and anisotropy, as a result of protein conformational changes. Due to the intricate nature of Trp fluorescence, data regarding the aforementioned fluorescence properties are frequently used in union to corroborate their individual results. Next, quenching of Trp fluorescence by exposure to internal or external quenchers is described with a discussion of the various mechanisms leading to fluorescence quenching. Resonance energy transfer is one of the mechanisms by which fluorescence quenching can occur, and the frequently used equations to derive crucial information from FRET experiments are presented. Here, we aim to give an overview of a recently-developed homogeneous assay that is based on FRET. Albeit that Trp has been used as an internal fluorophore in numerous FRET experiments, in this approach, coined as intrinsic Förster resonance energy transfer (iFRET), high affinity ligands conjugated to appropriate FRET acceptors are used in the detection of specific Trp expressing proteins. Here, FRET between active site Trp residues of native proteins and target-specific acceptor probes allow the label-free detection of these native proteins, circumventing cumbersome and expensive fluorescent labeling procedures. The iFRET technique holds high potential to detect specific target proteins in complex mixtures and even in non-engineered cells.

## 2. Tryptophan Fluorescence

**Figure 1:**
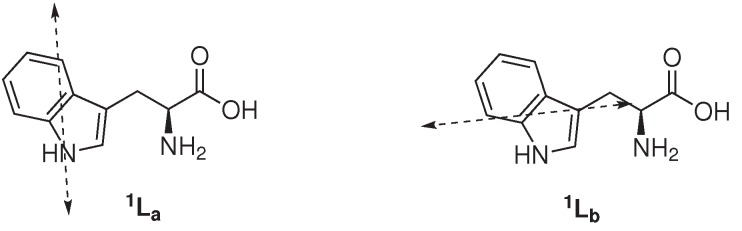
Electronic absorption transitions in tryptophan [2].

Amongst the three fluorescent amino acid constituents of proteins, Trp is the most abundant and is present at concentrations of about 1 mol % in soluble, cytoplasmic proteins and up to 3 mol % in membrane proteins [2]. The contribution of phenylalanine (Phe) to the intrinsic fluorescence of protein is negligible by virtue of its low absorptivity in addition to a very low quantum yield (Table 1). Although tyrosine (Tyr) has a quantum yield similar to Trp, the indole group of Trp is considered the dominant source of UV absorbance at *∼*280 nm and emission at *∼*350 nm in proteins [15]. Furthermore, in native proteins, Tyr emission is often quenched, presumably by its interaction with the peptide chain or via energy transfer to Trp [2,16]. Whereas Tyr may be regarded as a relative simple fluorophore, the spectroscopic properties of Trp are complex, in particular the high sensitivity to the (local) environment and its (at least) two different fluorescence lifetimes (*∼*0.5 and *∼*3.1 ns) [17,18]. Recent data indicate that the two fluorescence lifetimes are inherent to the Trp structure and independent of the excitation wavelength [19,20]. Several models have been proposed to elucidate the intricate (multi)exponential decay of Trp fluorescence. In one model it is assumed that the two different lifetimes are due to the emission from two nearly identical electronic absorption transitions (^1^*L_a_* and ^1^*L_b_* state, Figure 1) of Trp. The classic and general accepted rotamer model contends that structural heterogeneity (*i.e.*, the presence of rotameric structures about the C*_α_* – C*_β_* bond) in the ground state of Trp exist on a longer timescale (ms) in comparison to the excited state lifetime (ns) and give rise to the different fluorescence lifetimes. A third fluorescence lifetime of Trp is observed in some proteins (ranging from 6 to 9 ns in human and bovine serum albumins), and it is hypothesized that this is a result of the interactions of the surrounding with Trp.

**Table 1:** Fluorescence properties of aromatic amino acids in water at neutral pH [2,15,21].

	Lifetime (𝜏) (ns)		Absorption			Fluorescence
		λ (nm)	Absorptivity (*ϵ*)		λ (nm)	Quantum Yield (Φ_F_)
Tryptophan	3.1 (mean)	280	5600		348	0.2
Tyrosine	3.6	274	1400		303	0.14
Phenyl alanine	6.4	257	200		282	0.04

The photophysical properties of Trp are highly sensitive to its (local) environment. Current consensus is that Trp emission originates from only the ^1^*L_a_* state, except when the local environment is completely nonpolar. Therefore, the fluorescence of Trp is strongly affected by the polarity of its surrounding, promoting emission from both the ^1^*L_a_* and ^1^*L_b_* state. The large excited-state dipole moment of the ^1^*L_a_* state (*∼*6 Debye), in addition to its higher sensitivity towards hydrogen bonding, with respect to the excited ^1^*L_b_* state, results in its transition shift to lower energy [2,22]. As a consequence, Trp fluorescence maximum (λ_EM_) and intensity are highly influenced by the polarity of its micro-environment, hydrogen bonding and other non-covalent interactions, displaying a red shift in increasing polarity due to the emission of the ^1^*L_a_* state [23,24,25]. In hydrophobic environments, the ^1^*L_b_* state may have the lowest energy with respect to the ^1^*L_a_* state and dominate the emission of Trp, which would explain the blue shift of Trp fluorescence to increasing non-polar environments. Azurin, a native folded protein, where Trp-45 is embedded deep into a hydrophobic pocket, is a representable example of the least red-shifted Trp-containing proteins (λ_EM_*∼* 308 nm), comparable to skatole (3-methylindole, λ_EM_*∼* 310 nm) in cyclohexane [26,27]. In contrast, (denatured) proteins displaying solvent-exposed Trp residue(s), e.g., glucagon and melittin (λ_EM_*∼* 352 and 346 nm, respectively) are amongst the most red shifted.

In addition to the high sensitivity of Trp emission maximum and intensity to its local environment, Trp quantum yield (Φ_F_) can vary from 0.35 to 0.01 in different protein environments [28]. Interestingly, the quantum yield of skatole is *∼*0.3, regardless of the polarity of the solvent, being pure hydrocarbons or water [29]. The frequently proposed mechanism to explain the decrease in fluorescence quantum yield has been the electron transfer quenching by, for instance, the local peptide carbonyl group or by neighboring amino acid side chains [30,31,32]. The high dependency of Trp fluorescence on its surroundings have been exploited in numerous studies to elucidate, e.g., protein conformational changes and interactions with ligands. However, the interpretation of Trp spectroscopic data can be challenging when all of the different factors that can lead to the observed changes are taken into consideration.

### 2.1. Fluorescence Anisotropy

Fluorescence anisotropy measurements are based on the principle of photoselective excitation of fluorophores by polarized light (often) resulting in polarized emission [33]. The polarized emission is influenced by a number of processes, including motions that occur within the lifetime of the excited fluorophore, also known as rotational diffusion. Both intrinsic and extrinsic fluorophores can be used for fluorescence anisotropy measurements. A vast number of biological applications of fluorescence anisotropy have been reported to date due to the comparable timescale of rotational diffusion of biopolymers and the fluorescence lifetime of many fluorophores. Anisotropy measurement can provide information on the shape and size of proteins and have been used to measure protein-protein associations, the fluidity of membranes, binding and conformational dynamics [34,35,36,37,38,39,40,41,42,43]. However, fluorescence anisotropy is highly dependent on the environment (solvent viscosity), the size and shape of the fluorophore and the flexibility of the protein [2]. Anisotropy (*r*) is independent of the total sample intensity (*I*_T_, Equation (1)) and can be calculated according to Equation (2), where *I_‖_* is the intensity when the emission polarizer is orientated parallel (‖) to the to the polarized excitation. The intensity observed when the polarizer is perpendicular (⊥) to the excitation is given by *I*_⊥_.

(1) $$I_{Τ}=I_{∥}+2I_{⊥}$$

(2) $$r=\frac{I_{∥}-I_{⊥}}{I_{Τ}}$$

### 2.2. Fluorescence Quenching

Fluorescence quenching is an indispensable tool in protein research, since these studies are, in general, easy to perform, require only a small sample and are non-destructive. Quenching studies utilizing intrinsic Trp fluorescence of proteins can give a wealth of information regarding the location of the fluorophore within its macromolecular structure, thus providing structural information of the macro-molecule [44,45,46,47]. A variety of mechanisms, e.g., proton- and electron transfer, long-range energy transfer, induced conformational changes and various intramolecular reactions lay at the basis of Trp fluorescence quenching by external or internal ligands. The different mechanisms of quenching can be classified as dynamic quenching (collisional encounters) or static quenching (ground-state complex formation) between fluorophores and quenchers. In the case of collisional quenching, the dependence of the emission intensity on the quencher concentration (*Q*) is given by the well-known Stern–Volmer equation (Equation (3)). In this equation, *F*_0_ and *F* represent the fluorescence intensity, and 𝜏_0_ and 𝜏 are the lifetime in the absence (subscript 0) and presence of the quencher, respectively; and *K_sv_* is the Stern–Volmer constant.

(3) $$\frac{F_{0}}{F}=\frac{\tau_{0}}{\tau }=1+K_{sv}\cdot Q$$

Dynamic and static quenching can be distinguished by their different dependence on temperature and viscosity or, preferably, by lifetime measurements. Van de Weert *et al.* highlighted some pitfalls in the application of fluorescence quenching by external ligands [48]. It was proposed that the observed changes in fluorescence may arise due to an inner-filter effect and collisional (dynamic) quenching in addition to ligand binding (static quenching). Absorbance (or optical dispersion) of light at the excitation or emission wavelength is referred to as the inner-filter effect. Collisional quenching involves the contact of the excited fluorophore with ions or molecules that facilitate non-radiative transition to the ground state. External quenchers, such as molecular oxygen and paramagnetic moieties, are believed to induce rapid inter-system crossing of excited aromatic fluorophores by an electron spin exchange process, facilitating the conversion to the ground state. In the case of halogen ions and heavy atoms, the inter-system crossing via a spin-orbital coupling mechanism is promoted. Different types of quenchers can induce electron transfer to the excited state of the fluorophore. Amides, such as acrylamide, act as electron acceptors of the singlet state (of the excited aromatic fluorophore) [33]. Osysko *et al.* studied the electron transfer from the excited indole ring of Trp to one of the amides in the protein backbone, which is believed to be the major cause of fluorescence quenching by internal ligands [49]. Utilizing two model dipeptides, it was found that high pH results in a high quantum yield due to the low efficiency of the electron transfer event induced by the carboxylate ion, whereas low pH reduces the quantum yield by increasing the rate of electron transfer to the amide. Additionally, Chen *et al.* found that lysine and tyrosine side chains quench 3-methylindole (a representable model for Trp) fluorescence by excited-state proton transfer, and glutamine, asparagine, glutamic- and aspartic acid, cysteine and histidine side chains operate by an excited-state electron transfer mechanism [16]. Goldberg *et al.* reported the quenching of Trp and Tyr intrinsic fluorescence by thioamides in a distance-dependent manner [50]. The mechanism by which thioamides operate in fluorescence quenching is still uncertain; however, experimental data suggest an electron transfer mechanism. The probability of fluorescence quenching with external quenchers depends on the rate of collision of the quencher and the excited fluorophore (dynamic quenching). Therefore, it is expected that the fluorescence of Trp residues located on the surface of the protein will be influenced to a greater degree by the quencher, with respect to Trp residues embedded deep in the protein matrix. Fluorescence quenching can also arise via a resonance energy transfer mechanism, with a suitable acceptor molecule or metal ion. Cupredoxins, which possess a Trp residue located within 1 nm of the copper ion, e.g., azurin [51], stellacyanin [52] and amicyanin [53], exhibit the phenomenon of Trp fluorescence quenching upon metal ion binding via either Förster resonance energy transfer or an electron transfer mechanism [54,55].

### 2.3. Selected Examples of Tryptophan Fluorescence in the Elucidation of Protein Structures

The dependency of Trp fluorescence properties (e.g., absorption and emission maxima, fluorescence intensity, anisotropy and quantum yield) on its micro environment has been exploited in numerous studies to derive inference with respect to protein conformational changes and/or protein denaturation [14,56,57,58,59,60,61]. Gorinstein *et al.* utilized the change in intrinsic Trp fluorescence and surface hydrophobicity of human serum proteins to evaluate the effect of beer consumption on protein integrity in patients suffering from coronary artery disease [62]. A slightly red-shifted fluorescence (λ_EM_), a decrease in fluorescence intensity and surface hydrophobicity were observed in human serum albumin and human serum globulin after 30 days of exposure of patients to moderate alcohol consumption. The altered fluorescence properties of these human serum proteins were attributed to structural disruption of the proteins in addition to a change in their compactability, as a result of ethanol consumption.

The denaturation of bovine serum albumin (BSA) under the influence of sodium dodecyl sulfate (SDS) at various pH values was investigated based on fluorescence anisotropy measurement of tryptophan, in addition to its fluorescence quenching [63]. BSA, a globular protein with a molecular weight of 64 kDa and an isoelectric point (pI) of 4.9, is involved in the transportation of physiological metabolites. BSA contains two tryptophan residues, and their fluorescence is quenched in the presence of ionic detergents, such as SDS, which is associated with the denaturation of BSA. The observed stepwise quenching of BSA intrinsic fluorescence in addition to a gradual increase in the degree of anisotropy (derived from Equation (2)) indicated a two-stage denaturation process. The first stage involved the loosening of protein globules, and the second stage is complete unfolding of the protein. It was found that at pH values lower than the pI, the generally positive-charged BSA (5 µM) binds strongly to the SDS anions, resulting in strong BSA fluorescence quenching. In contrast to the aforementioned, at pH values higher than the pI, only weak quenching of BSA fluorescence was observed. These findings were corroborated by a higher increase in the degree of BSA fluorescence anisotropy at pH values lower than the pI in comparison to pH values higher than the pI.

Besides native Trp containing proteins, e.g., azurin, bovine and human serum albumin, site-directed mutagenesis enables the introduction (or deletion) of Trp residues into proteins. However, the alteration of Trp residues in proteins can influence its shape and function. This approach was employed by Kozachkov *et al.*, who observed two essential conformational changes in the Na^+^ /H^+^ antiporter NhaA based on Trp fluorescence [64]. NhaA plays a crucial role in maintaining pH and Na^+^ homeostasis in *Escherichia coli* and other enterobacteria and, possibly, *Homo sapiens*. Native NhaA contains eight Trp residues, which result in high background fluorescence. Therefore, site-directed mutagenesis was employed to generate single Trp mutants in addition to a Trp-less variant of NhaA. Two single tryptophan variants, Trp/F136W and Trp/F339W, grown on selective media, were found to have antiport activities that are similar to the Trp-less NhaA. Fluorescence studies with the single Trp/F136W mutant revealed that a pH shift from pH 6.0 to 8.5 induces a red shift in Trp emission. Furthermore, a dramatic increase in fluorescence, which was reversible, was observed, and the addition of either Na^+^ or Li^+^ had no effect. In contrast, the single Trp/F339W mutant remained inert to pH changes; however, the addition of either Na^+^ or Li^+^ drastically resulted in fluorescence quenching at alkaline pH.

## 3. Förster Resonance Energy Transfer (FRET)

Förster resonance energy transfer (FRET) is a non-radiative energy transfer process (without the transfer of a photon) between a donor fluorophore in the the excited state and a acceptor molecule in the ground state. This long-range (1–10 nm) dipole-dipole coupling has emerged as a powerful technique to unravel the dynamics of biomolecules [25]. Numerous approaches to utilize FRET in protein studies have been developed over the past few years [65,66,67,68,69]. Several difficulties are encountered when using FRET in biological systems, for example the measured fluorescence intensities must be corrected for the auto-fluorescence of cells, and in most cases, the contribution from unbound fluorophores results in poor sensitivity, which presents difficulties in the interpretation of the results. Several factors influence the rate of resonance energy transfer, e.g., the extent of spectral overlap of the emission spectrum of the donor with the absorption spectrum of the acceptor, the quantum yield of the donor, the relative orientation of the donor and acceptor transition dipoles and the distance between the donor and acceptor molecules [2]. Besides the favorable distance (which is usually the size of a protein or the thickness of a membrane), several criteria have to be fulfilled for successful application of FRET in biological systems. For instance, the FRET acceptor should be inert to the excitation wavelength of the FRET donor (in other words, the acceptor should have an absorption minimum at the donor excitation maximum). Another important characteristic is the quantum yield of the FRET acceptor. The rate of FRET transfer is inversely proportional to the sixth power of the distance between the fluorophore and acceptor (Equation (4)).

(4) $$k_{T}=\left(\frac{1}{\tau_{D}}\right)\cdot {\left(\frac{R_{o}}{R}\right)}^{6}$$

(5) $$R_{0}=0.211\cdot {\left\{\kappa^{2}\cdot n^{-4}\cdot \Phi_{D}\cdot J\right\}}^{\frac{1}{6}}$$

In this equation, represents the fluorescence lifetime of the donor, *i.e.*, the measured lifetime in the absence of an 𝜏Dacceptor, *R* is the distance between the donor and acceptor and *R*_0_ is the distance at which 50% of the donor excited state energy is transferred to the acceptor [11,70]. The distance parameter *R*_0_ can be estimated based on the spectroscopic and mutual dipole orientation parameters between the donor and acceptor (Equation (5)) and is expressed in nanometers (nm). In this expression, *κ* is a dimensionless factor ranging from zero to four and depends on the relative orientation of the dipole moments with respect to the donor emission and the acceptor absorption and with respect to the axis connecting donor and acceptor. Head to tail parallel transition dipoles (*→ ·· →*) give a value of four for *κ*^2^, and for parallel oriented dipoles (*↑ ·· ↑*) *κ*^2^ is one. However, when the orientation of the dipoles are perpendicular (*↑ ··*↙), *κ*^2^ equals zero, causing serious calculation errors. In general, *κ*^2^ is assumed to be 23 in the calculation of *R*_0_, which holds true for randomized orientation of donor and acceptor dipoles as a result of rotational diffusion proceeding energy transfer. In the case of a range of static orientations of donor and acceptor during the fluorescent lifetime, *κ*^2^ may be assumed to be 0.476 [2]. To minimize calculation errors in determining *R*_0_, the limit of *κ*^2^ can be obtained from fluorescence anisotropy measurements of the donor and acceptor. At present, X-ray crystallography and NMR spectroscopy are the only methods available to measure *κ*^2^; however, these methods render distance determination by FRET redundant. The refractive index of the corresponding medium is given by *n*, and Φ_D_ is the quantum yield of the donor. For most FRET donor/acceptor pairs, the distance *R*_0_ is in the 1–7 nm range.

(6) $$J=\epsilon_{A}\frac{\int_{0}^{\infty }f_{D}(\lambda )f_{A}(\lambda )\lambda^{4}d\lambda }{\int_{0}^{\infty }f_{D}(\lambda )d\lambda }$$

(7) $$E=1-\frac{\tau_{DA}}{\tau_{D}}=1-\frac{I_{DA}}{I_{D}}=\frac{1}{1+{\left(R/R_{0}\right)}^{6}}$$

The degree of spectral overlap between donor emission and acceptor absorption (*J*) is expressed in units of *M^−^*^1^*·* *cm^−^*^1^*·* *nm*^4^ (Equation (6)). Here, ϵ_A_ is the acceptor molar extinction coefficient at its absorption maxima and is expressed in units of *M^−^*^1^*·* *cm^−^*^1^. The donor emission spectrum (*f*_D_(λ)) and the normalized acceptor spectrum (*f*_A_(λ)) are dimensionless. As a result of FRET, the fluorescence lifetime of the donor is reduced. Therefore, FRET efficiencies (*E*) may be determined from the decrease in the lifetime (𝜏), the corresponding decrease in the intensity of fluorescence (*I*) in the presence and absence of the acceptor and from the Förster distance (*R*_0_, Equation (7)). When FRET experiments are conducted with a fluorescent acceptor, the relative FRET efficiency (*E_rel_*.), using the ratio between donor and acceptor emission, can be calculated according to Equation (8), which is known as the ratiometric method. Here, *I*_D_ and *I*_A_ are the total fluorescence intensities of the donor and acceptor, respectively, following excitation of the donor. This method can be used to monitor relative changes of the FRET efficiency and is usually used in kinetic measurements

(8) $$E_{rel.}=\frac{I_{A}}{I_{D}+I_{A}}$$

(9) $$\Phi =\Phi_{R}\frac{I}{I_{R}}\frac{OD_{R}}{OD}\frac{n^{2}}{n_{R}^{2}}$$

The quantum yield (Φ_F_) of a fluorophore can be estimated by comparison with standards of known quantum yields. The quantum yields of standards are often independent of their excitation wavelength. Determination of the quantum yield of an unknown fluorophore is in general accomplished by comparison of its wavelength integrated intensity to that of the standard. Equation (9) can be used to calculate the quantum yield of a fluorophore, in which *I* represents the integrated efficiency, *OD*is the optical density and *n* is the refractive index, and the reference fluorophore is indicated by subscript R.

One approach to utilize FRET is in the development of fluorescence immunoassay (FIA). In FIA, the amino acid residue(s) of target proteins are labeled with either a FRET acceptor or donor, with preference for the latter, in combination with a target specific antibody (or high affinity ligand), which is labeled with the complementary FRET donor or acceptor. Alternatively, two antibodies with selectivity for different epitopes on the protein surface can be used in sandwich-type fluorescence immunoassay. Labeling of both the antibodies with either a FRET donor or acceptor would result in FRET fluorescence upon binding of both antibodies to the protein [71]. However, the labeling of biopolymers, e.g., proteins and antibodies, with either a FRET donor or acceptor can be cumbersome and expensive and could induce conformational and functional changes of the targets, resulting in aberrancy.

## 4. Intrinsic Förster Resonance Energy Transfer

**Scheme 1:**
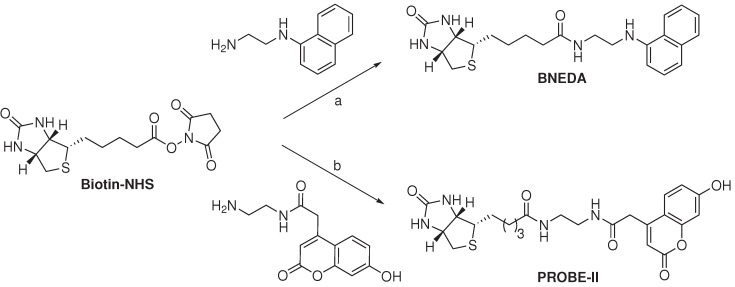
Synthesis of FRET acceptor biotin probes for the detection of streptavidin [75,77]. Reagent and conditions: (**a**) CH_2_Cl_2_, 12 h in the dark, about 50% from biotin; (**b**) Et_3_N, DMF, 5 h, 63%.

**Figure 2:**
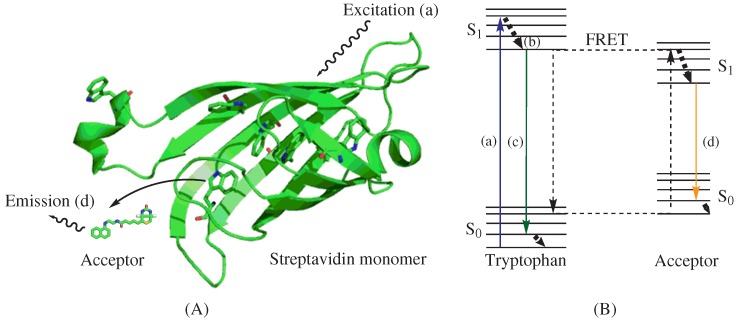
Illustration and Jablonski diagram of the intrinsic Förster resonance energy transfer technique. (**A**) A model structure of biotin probe BNEDA with a streptavidin monomer, which was generated from a crystal structure of streptavidin (PDB ID: 1STP) by PyMol; and (**B**) A Jablonski diagram illustrating intrinsic FRET; (**a**) Excitation of tryptophan at 280 nm; (**b**) Vibrational relaxation; (**c**) Emission of tryptophan near 350 nm; (**d**) Emission of biotin-fluorophore conjugate biotin-naphthyl conjugate (BNEDA) at 430 nm.

The use of Trp intrinsic fluorescence as a FRET donor can circumvent tedious fluorescence labeling of target proteins [72,73,74]. Furthermore, utilizing the intrinsic protein fluorescence of Trp residues allows the development of homogeneous assays to, for instance, assess protein levels and ligand binding. To this end, a FRET technique, coined iFRET, which utilizes the intrinsic Trp fluorescence of proteins in conjunction with suitable acceptor fluorophores, was developed [75,76,77]. In this approach, the Trp residues at the binding site of proteins and target-specific ligand-fluorophore conjugates act as the FRET donor and acceptor, respectively. Liao *et al.* utilized a biotin-naphthyl conjugate (BNEDA, Scheme 1) to achieve the close proximity required for FRET between the Trp residues near the biotin-binding site of streptavidin (STV) and the naphthylamine fluorophore [75]. This FRET approach is illustrated in Figure 2A and depicts an STV monomer and the BNEDA probe. In addition, the Jablonski diagram in Figure 2B illustrates the electronic states of tryptophan, the FRET donor, and the BNEDA probe, which serves as the FRET acceptor. STV contains six Trp residues near the ligand-binding site, and STV homo-tetramers can bind four biotin derivatives with an unusually strong binding affinity (*K*_D_*∼* 10*^−^*^14^ M [78]).

**Table 2:** Intrinsic tryptophan fluorescence as the FRET donor with the BNEDA probe [75].

	Excitation	Emission	Stokes Shift	Quantum Yield (Φ_F_)
	(nm)	(nm)	(nm)	
Streptavidin	280	340	60	0.2 *
BNEDA	325	430	105	0.52
Streptavidin-BNEDA	280	430	150	0.52

* Quantum yield of tryptophan.

The BNEDA probe was prepared from biotin-*N*-hydroxysuccinimide (biotin-NHS) and *N*-(1-naphthyl)ethylenediamine in about a 50% yield. The unbound BNEDA probe was found to produce negligible emission at 430 nm, when excited at 280 nm. Furthermore, the BNEDA probe had an additional absorbance peak around 245 nm, which was five- and seven-times lower than the absorbance at 325 and 280 nm, respectively. The quantum yield (Φ_F_) of BNEDA was calculated via Equation (9) and was found to have a ratio of 2.62 *±* 0.02 (*n* = 2) to that of Trp. Excitation of STV at 280 nm produced strong fluorescence at 340 nm and negligible emission at 430 nm (Table 2). When the BNEDA probe was incubated with STV for 2 min, high and stable fluorescence was observed at 430 nm when excited at 280 nm. Excitation of the complex of STV and the BNEDA probe at 280 nm showed an 80% decrease in fluorescence at 340 nm with respect to STV alone. Fluorescence studies with the BNEDA probe, conducted in the presence of free Trp (at levels equivalent to its occurrence in STV) instead of STV, showed no emission reduction at 340 nm nor an increase of fluorescence at 430 nm. In competition experiments, executed with a mixture containing proteins from cell lysates of *E. coli*, STV and BNEDA, the fluorescence at 430 nm was reduced by the addition of free biotin (concentration dependent). The BNEDA probe was found to have a similar binding affinity (*K*_D_ = 1*.*6 *·* 10*^−^*^14^ M) as biotin to STV. The limit of detection of an STV subunit with the BNEDA probe, in the presence of proteins from *E. coli* cell lysates (2.0 mg/mL), was found to be *∼* 0.15 nM. 

The iFRET fluorescence from the BNEDA probe (*λ_max_* = 430 nm) is very close to the auto-fluorescence of cells when irradiated at 280 nm, which may hamper *in vivo* application thereof. It was shown that the background fluorescence remained consistent in the presence of substances such as MeOH, DMSO, MgCl_2_, NaCl, NaN_3_, glycerol and proteins from *E. coli* cell lysates (2.0 mg/mL). In addition, the relative short Stokes shift of the BNEDA probe during FRET experiments (90 nm) may induce self-absorption, which in turn would decrease the accuracy of the measurement. With this rationale in mind, Kim and co-workers developed coumarin derivatives as efficient FRET acceptors for Trp emission [77]. The most promising iFRET probe, PROBE-II, comprises biotin (*i.e.*, as a ligand for STV) conjugated via a linker to a fluorescent coumarin derivative and was obtained in 63% yield from biotin-NHS (Scheme 1).

When PROBE-II was irradiated with UV-light (280 nm), corresponding to STV excitation wavelength, low fluorescence was detected at 460 nm. Illumination of PROBE-II with near-UV-light (360 nm), corresponding to its absorption maxima, resulted in strong fluorescence at 460 nm (Table 3). The quantum efficiency (Φ_F_) of PROBE-II, with umbelliferone as the standard (Φ_F_ = 0.7), was found to be 0.47. iFRET experiments were conducted in the presence of STV, and bovine serum albumin (BSA) was used as a negative control to gain insight into the selectivity of the iFRET probe. BSA was selected as the negative control due to the presence of two Trp residues in its sequence; however, BSA has no affinity towards biotin. Spectroscopic data revealed the characteristic FRET phenomenon when PROBE-II was used in the presence of native STV. The FRET efficiency was calculated according to Equation (7) and was found to be 0.14. The Förster distance was derived from Equations (4) and (5) and was calculated to be 2.04 nm. On the contrary, FRET signals were absent when PROBE-II was evaluated in the presence of either BSA or denatured STV.

**Table 3:** Intrinsic tryptophan fluorescence as the FRET donor with coumarin-based PROBE-II [77].

	Excitation (nm)	Emission (nm)	Stokes Shift (nm)
Streptavidin	280	340	60
PROBE-II	340	460	120
Streptavidin-PROBE-II	280	460	180

Recently, Zhang *et al.* reported the evaluation of a small library of known fluorophores (Table 4) as FRET acceptors for Trp and Tyr residues [79]. The selected fluorophores all have excitation wavelength maxima around 335 nm, corresponding to Trp and Tyr emission. The properties of interest included the rotational freedom (the angular factor that influences FRET efficiency), the quantum yield of the bound fluorescent probes and the degree of spectral overlap between protein fluorescence and the excitation wavelength of the fluorophores. Additional factors of consideration are the ease of synthesis, cost and stability of the corresponding iFRET probes. First, the excitation and emission maxima of the fluorophores were examined in mixtures of THF (0 to 15 vol %) in neutral phosphate buffer. It was postulated that these conditions mimic enzyme binding sites based on previous observations that a final concentration of 15 vol % THF in neutral phosphate buffer caused a blue-shift in the emission maxima of the free BNEDA probe (Scheme 1), comparable to the blue shift resulting from the binding of the probe to STV. A selection of the reported findings is presented in Table 4, showing the emission and excitation maxima, the quantum yield (Φ_F_) and Stokes shift of the tested library of fluorophores in the presence (15 vol %) and absence of THF. The dansyl fluorophore (**D**) exhibits the most blue-shifted emission under the influence of THF, whereas 8-hydroxyquinine (**B2**) and the coumarin derivatives, **C1** and **C2**, revealed only minor spectral changes. The naphthyl derivatives, **A1** and **A2**, gave the highest quantum yield, which was shown to increase by the addition of THF. The difference in excitation around 280 and 335 nm was analyzed and based on those ratios; in addition to the other fluorescent properties, 1-naphthylamine (**A1**) was deemed most suitable as the FRET acceptor and the coumarin derivative **C1** the second best. Considering the synthetic challenges associated with the derivatization of **C1**, it was decided to exclude coumarin derivatives from further assessment. Conjugates of 1-naphthyl amine (**A1**), dansyl amide (**D**) and acridine-9-carboxyl acid (**E2**) with target-specific ligands were further evaluated as iFRET probes.

**Table 4:** Small library of fluorophores as the FRET acceptors in conjunction with tryptophan as the FRET donor and their fluorescence properties at different levels of THF in neutral phosphate buffer [79]. 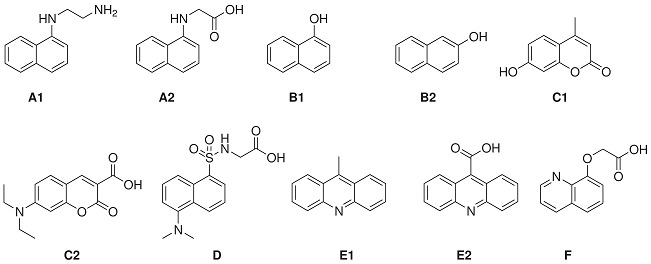

	Excitation (nm)		Emission (nm)		Stokes Shift (nm)		Quantum Yield (Φ_F_)	
% THF	0	15	0	15	0	15	0	15
**A1**	325	330	443	431	118	101	0.71	0.89
**A2**	324	325	447	439	123	114	0.68	0.81
**B1**	293	296	468	466	175	173	0.12	0.15
**B2**	324	324	411	407	87	83	0.24	0.25
**C1**	323	323	451	451	128	128	0.45	0.37
**C2**	384	385	481	473	97	88	0.10	0.16
**D**	327	329	562	551	235	222	0.04	0.15
**E1**	357	357	436	433	79	76	0.17	0.25
**E2**	354	356	448	430	94	74	0.35	0.23
**F**	309	306	491	488	182	182	0.04	0.08

**Scheme 2:**
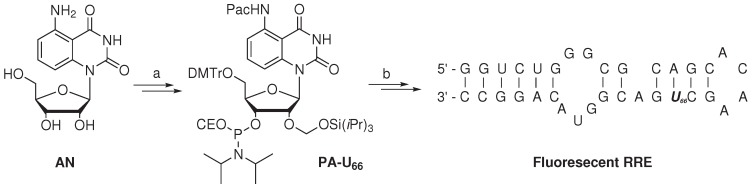
Synthesis of a fluorescent (oligo)nucleotide as the FRET acceptor with tryptophan as the FRET donor [76]. Reagent and conditions: (**a**) Six steps, 9% overall yield; (**b**) Standard solid-phase RNA synthesis.

The study of RNA binding proteins, which often displays Trp residues at the RNA binding site, via the iFRET technique was reported by the group of Xie [76]. To minimize the disturbance of RNA-protein interactions by the use of classic fluorophores, a fluorescent nucleoside derivative (**AN**, Scheme 2) was utilized. The absorptivity of nucleoside **AN** at 280 nm (*i.e.*, the λ*_max_* of Trp) was found to be minimal, whereas its absorption at 350 nm overlaps with the emission of Trp, producing fluorescence at 440 nm. A FRET efficiency of 0.42 *±* 0.04 suggested excellent FRET pairing of **AN** with Trp. The critical Förster distance was experimentally determined to be 2.2 nm, which is a proper distance to monitor RNA-protein binding events.

Artificial nucleoside **AN** was converted in six steps with an overall yield of 9% to the 3’-phosphoramidite nucleotide **PA-U**_66_, rendering it suitable for usage in standard solid-phase RNA synthesis. The artificial fluorescent nucleotide was utilized to investigate the binding of the Rev protein, a key HIV-1 regulatory protein, with the Rev responsive element (RRE), its cognate RNA target. The Rev protein is involved in the transport of immature viral mRNA from the nucleus to the cytoplasm of the host cell. The Rev protein specifically binds RRE with high affinity, which has been attributed to the arginine-rich domain of the Rev and Stem-loop IIB of the RRE. The Rev peptide (amino acid sequence: DTRQARRNRRRR**W**_45_RERQRAAAAR) contains a single Trp (W_45_) residue, which is embedded within the RNA binding domain. Uridine nucleotide (U_66_) of the RRE, positioned in the vicinity of the Rev binding site, was selected to incorporate the developed fluorescent nucleotide **PA-U**_66_. The modified 34-mer RRE oligonucleotide was prepared via standard solid-phase oligonucleotide synthesis, utilizing phosphoramidite chemistry. After purification (by PAGE) and analysis (by MALDI-TOF mass spectrometry), the modified, folded RRE oligonucleotide containing the fluorescent nucleobase was found to have a similar melting temperature (*T_m_* = 66 *±* 1 *°*C) with respect to unmodified RRE (*T_m_* = 68 *±* 1 *°*C). The similar melting temperature indicates that the two oligomers are of comparable stability, which suggests that the incorporation of the fluorescent nucleobase resulted in minimal perturbation. A lower quantum yield was observed (emission at 440 nm) for the fluorescent RRE construct in comparison to parent nucleoside **AN**, when excited at 350 nm.

When the Ret protein was titrated into the modified RRE oligonucleotide, excitation at 280 showed a continues decrease of emission around 350 nm and an increase of fluorescence at 440 nm (Table 5). The binding constant (*K*_D_ = (7 *±* 5)*×*10*^−^*^8^ M) of the fluorescent RRE ribonucleotide to the Ret protein was derived from FRET efficiencies and is in agreement with literature values using unmodified RRE. Furthermore, fluorescent anisotropy measurement revealed similar affinity of the Ret protein to both the fluorescent (*K*_D_ = (2 *±* 1) *×* 10*^−^*^8^ M) and unmodified RRE (*K*_D_ = (3 *±* 2) *×* 10*^−^*^8^ M) with respect to FRET-derived binding affinities. Additionally, it was demonstrated that the FRET system gives information about displacement of the fluorescent RRE oligonucleotide with a competing peptide, the RSG peptide (amino acid sequence: DRRRRGSRPSGAERRRRRAAAA), lacking Trp residues, with a higher binding affinity for the RRE oligonucleotide. Titration of the RSG peptide into a solution of the Rev peptide bound to the fluorescent RRE oligonucleotide revealed a decrease in fluorescence at 440 nm. The inhibition concentration (*IC*_50_ = (2 *±* 1) *×* 10*^−^*^6^ M) of the RSG peptide with the fluorescent RRE oligonucleotide confirmed it to be a better binder with respect to the Rev peptide.

**Table 5:** Intrinsic tryptophan fluorescence as the FRET donor with fluorescent (oligo)nucleotide [76].

	Excitation (nm)	Emission (nm)	Stokes Shift (nm)
Ret protein	280	350	70
Nucleotide **AN**	350	440	90
Ret protein-nucleotide **AN**	280	440	160
Ret protein-fluorescent RRE	280	440	160

In the development of biosensors, Zhang and coworkers reported a selective and sensitive homogeneous assay of serum albumin with 1-anilinonaphthalene-8-sulfonate (ANS), based on the iFRET technique [80]. Serum albumin is an important biomarker for diseases related to kidney and liver function, and ANS is a known, nonspecific ligand thereof with moderate affinity [81,82]. The spectroscopic properties of ANS are complex, having two characteristic near-UV absorption maxima at 352 and 375 nm and an additional excitation peak near 270 nm, which essentially renders it unsuitable to use as a FRET acceptor for Trp. The fluorescence of ANS originates from two different excited states: the initial excited state, localized on the naphthalene ring, emits around 470 nm in nonpolar solvents. In polar solvents (e.g., methanol, ethanol), the initial excited state undergoes an intramolecular electron-transfer reaction, resulting in fluorescence at longer wavelengths. In phosphate buffer, serum albumin (200 mM) and ANS (300 nM) showed negligible emission at 460 nm upon excitation at 280 and 350 nm. Interestingly, the mixture of serum albumin with increasing concentrations of ANS revealed a continuous increase in fluorescence at 470 nm and a decrease in emission at 350 nm when excited at 280 nm. These results confirmed the occurrence of FRET between ANS and the excited Trp residues of serum albumin, with a nearly 52-fold increase in quantum yield with respect to free ANS in neutral phosphate buffer, when excited at 280 nm. With the developed assay, serum albumin was quantified from 1.8 to 25 nM, the lower lower limit of which corresponded to less than 1% that of the bromocresol green assay. Furthermore, other globular proteins gave negligible signals, and interfering substances present in clinical sera are well tolerated.

## 5. Concluding Remarks

Fluorescence spectrometry has become a standard tool in many areas of research and represents a versatile alternative for classic studies employing radioactive labels. A plethora of fluorescent techniques and methods have been developed to study biological events. The complex nature of biological systems has facilitated the development and exploration of several label-free approaches. Especially the utilization of natural fluorophores, such as the fluorescent amino acid residues, are gaining widespread applications. The dependence of Trp fluorescent properties on its micro-environment has enabled the study of different facets of proteins. Methods based on the changes in Trp absorption and emission maxima, fluorescence intensity and anisotropy have proven invaluable in protein research. FRET is a powerful technique to gain specific and real-time information of biological processes. The use of the intrinsic fluorescence of target proteins, originating from Trp residues, as the FRET donor in conjunction with target-specific fluorescent probes as the complementary FRET acceptor is a valuable addition to the toolbox. This technique holds true value, e.g., in drug discovery approaches (high throughput screening), (bio)imaging and in the development of biosensors.
